# Poly[[[μ-1,4-bis­(pyridin-4-ylmeth­yl)piperazine][μ-4-(2-carboxyl­atoeth­yl)benzoato]copper(II)] monohydrate], a coordination polymer with twofold inter­penetrated cds topology networks

**DOI:** 10.1107/S2414314623008556

**Published:** 2023-10-03

**Authors:** Gabrielle J. Gaskin, Robert L. LaDuca

**Affiliations:** a E-35 Holmes Hall, Michigan State University, Lyman Briggs College, 919 E. Shaw Lane, East Lansing, MI 48825, USA; Vienna University of Technology, Austria

**Keywords:** crystal structure, copper, coordination polymer, disorder, tri-periodic network

## Abstract

The title compound, {[Cu(C_10_H_8_O_4_)(C_16_H_20_N_4_)]·H_2_O}_*n*_, contains square-planar coordinated Cu^II^ ions linked by 4-(carboxyl­atoeth­yl)benzoato (ceb) and 1,4-bis­(pyridin-4-ylmeth­yl)piperazine (bpmp) ligands into a tri-periodic coordination polymer with twofold inter­penetrating 6^5^8 **cds** topology. Positional crystallographic disorder among the copper atoms, the ethyl­carb­oxy group of the ceb ligands, and the water mol­ecules of crystallization exists in a refined 0.655 (6)/0.345 (6) ratio.

## Abstract 2

A divalent copper tri-periodic coordination polymer with twofold inter­penetrating 6^5^8 **cds** topology, {[Cu(ceb)(bpmp)]·H_2_O]_*n*_, was structurally characterized by single-crystal X-ray diffraction.

## Graphical abstract

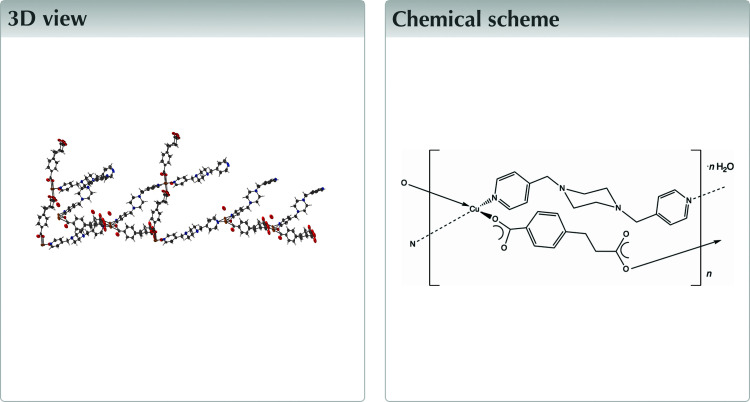

## Structure description

Our group has employed 1,4-bis­(pyridin-4-ylmeth­yl)piperazine (bpmp) in the generation of divalent metal coordination polymers with intriguing di-periodic and tri-periodic network topologies (Robinson *et al.*, 2015 ▸). For example, the copper oxalate (ox) bpmp-containing phase {[Cu_2_(ox)_2_(bpmp)]·6H_2_O}_*n*_, manifests a unique tri-periodic structure with a (5^3^8^3^)_2_(5^4^8^2^) self-penetrating network. Use of oxy(bis­)benzoate (oba) with bpmp generated {[Co_3_(oba)_3_(bpmp)_2_]_*n*_, which exhibits a highly self-entangled tri-periodic network with 4^4^5^17^6^7^ topology (Martin *et al.*, 2008 ▸). The title compound was isolated during an attempt to prepare a divalent copper coordination polymer containing both bpmp and 4-(carboxyl­atoeth­yl)benzoato (ceb) ligands.

**Figure 1:**
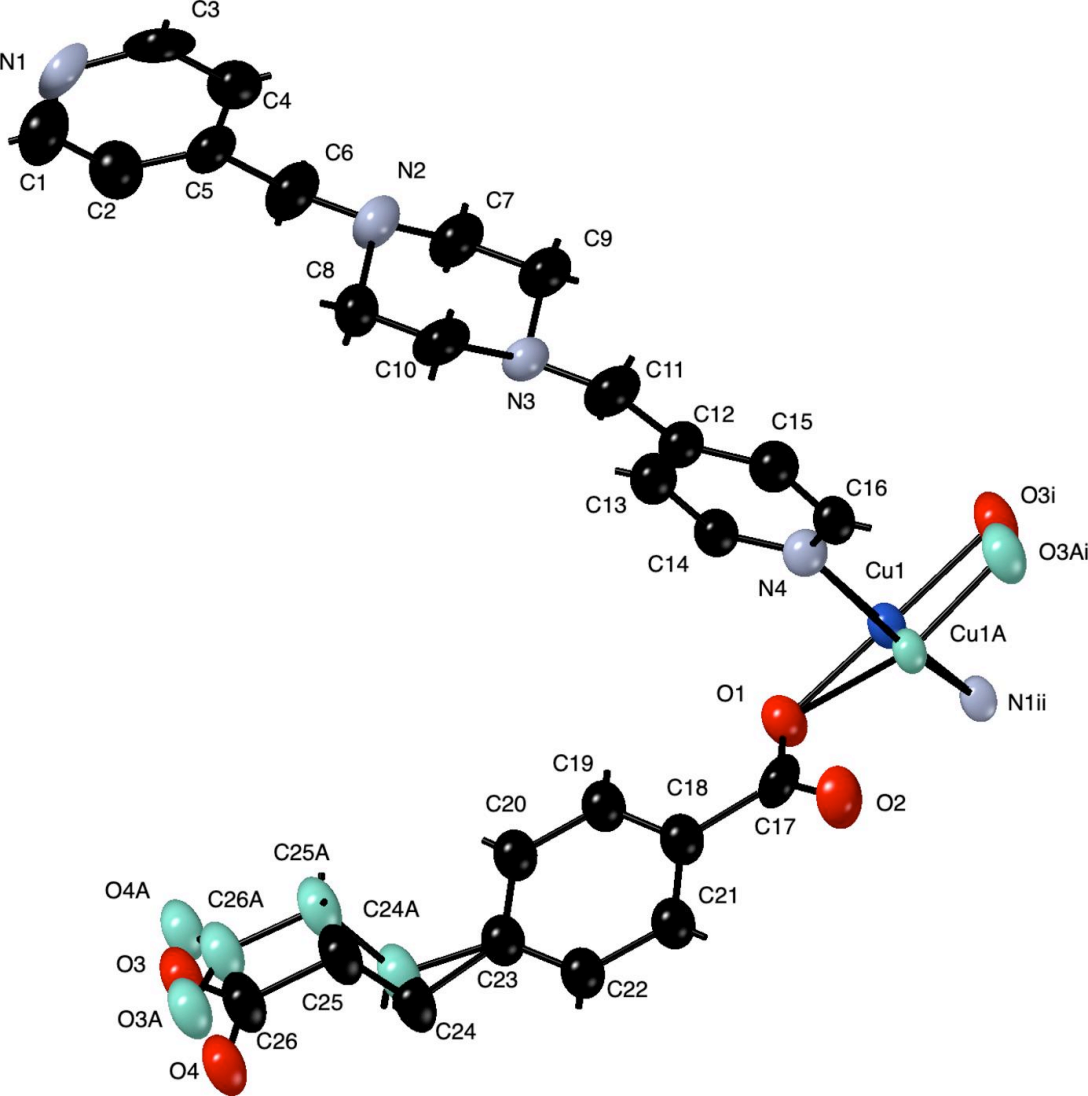
Copper coordination environment in the title compound with full ceb and bpmp ligands. Displacement ellipsoids are drawn at the 50% probability level. Color code: Cu, dark blue; O, red; N, light blue; C, black. The minor disorder components are shown in teal. H-atom positions are shown as sticks. Symmetry codes are as listed in Table 1 ▸.

The asymmetric unit of the title compound contains a Cu^II^ atom disordered over two positions, a fully deprotonated ceb ligand whose carboxyl­atoethyl group is disordered over two sets of sites, a bpmp ligand, and two disordered water mol­ecules of crystallization. All disordered parts in the crystal structure are present in a refined ratio of 0.655 (6):0.345 (6). The Cu^II^ atom is coordinated in an {N_2_O_2_} square-planar fashion by two *trans*-oriented pyridyl N-atom donors from two bpmp ligands, and two *trans*-oriented carboxyl­ate O-atom donors from two ceb ligands (Fig. 1 ▸). Pertinent bond length and angle information for the coordination sphere is listed in Table 1 ▸.

**Figure 2:**
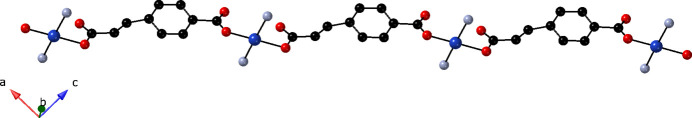
[Cu(ceb)]_*n*_ coordination polymer chain in the title compound. Only the major disordered components within the ceb ligands are shown, and only the major disorder atom Cu1 is shown.

**Figure 3:**
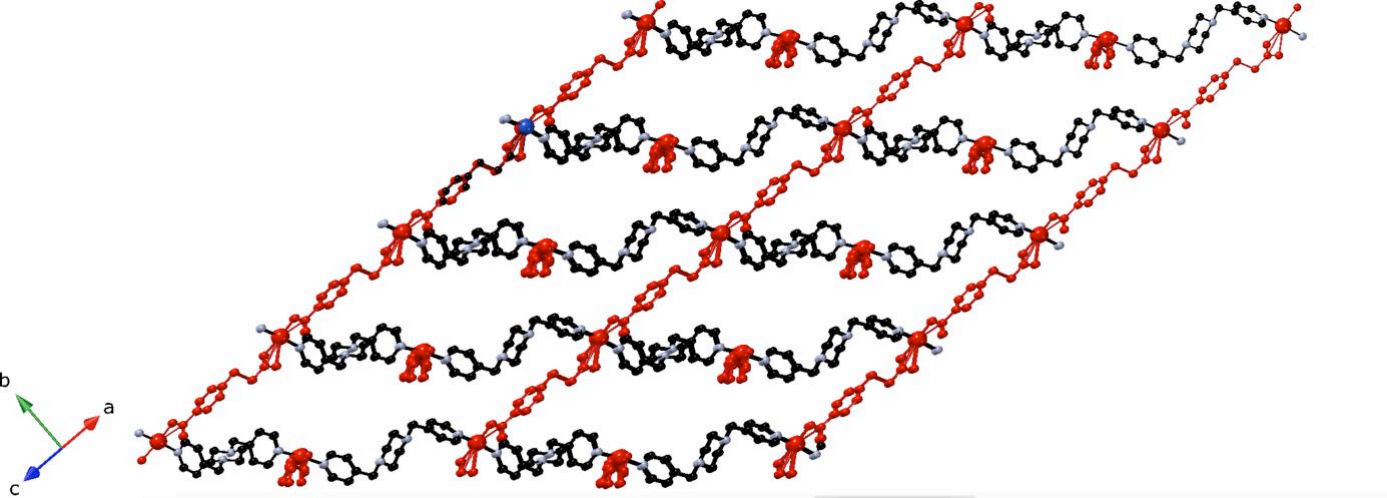
A [Cu(ceb)(bpmp)]_*n*_ **cds** coordination polymer network in the title compound. The [Cu(ceb)]_*n*_ coordination polymer chains are depicted in red.

**Figure 4:**
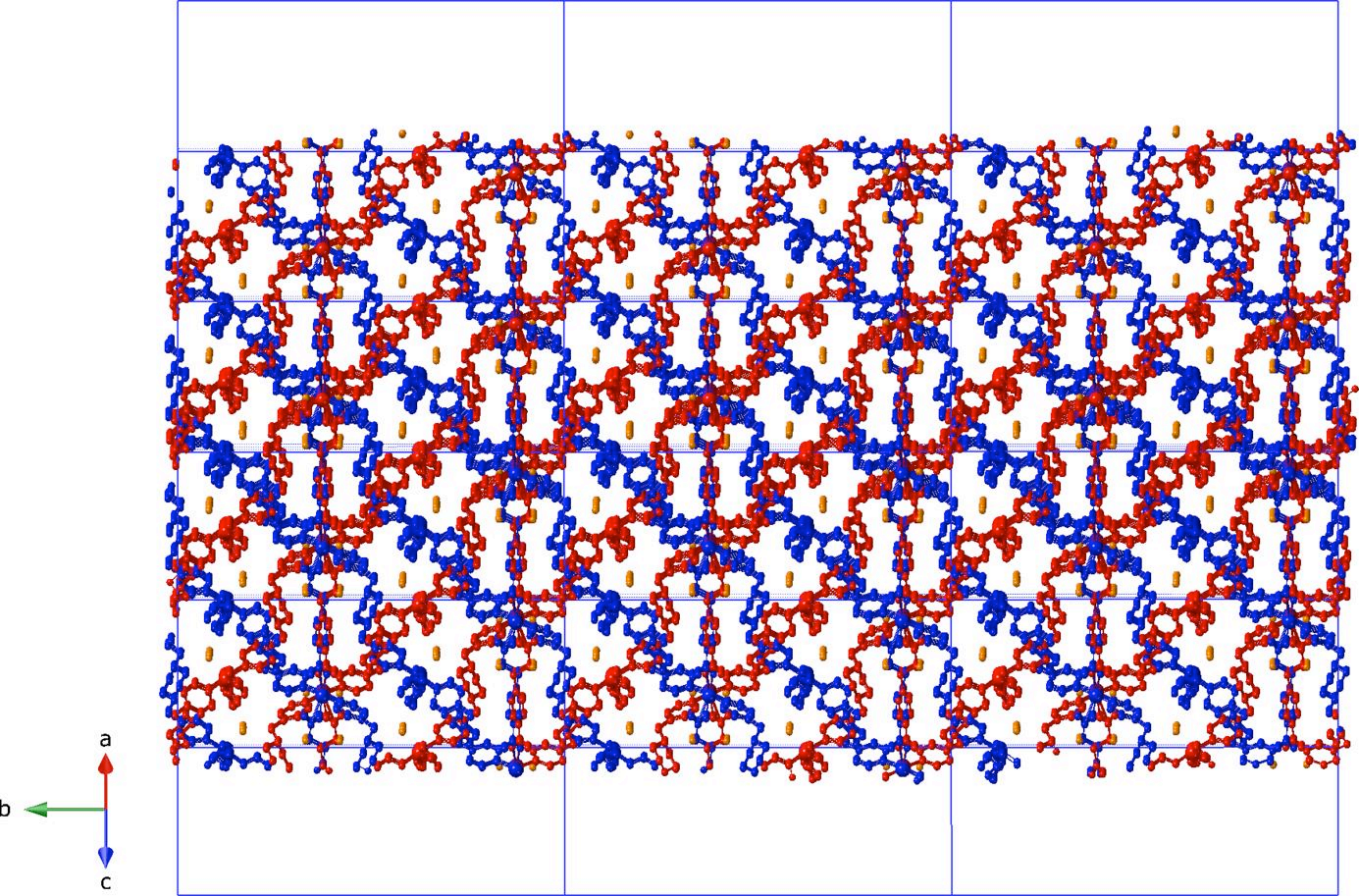
Twofold inter­penetration of [Cu(ceb)(bpmp)]_*n*_ tri-periodic coordination polymer networks in the title compound. Each network is shown in a different color. Unit-cell outlines are shown.

**Figure 5:**
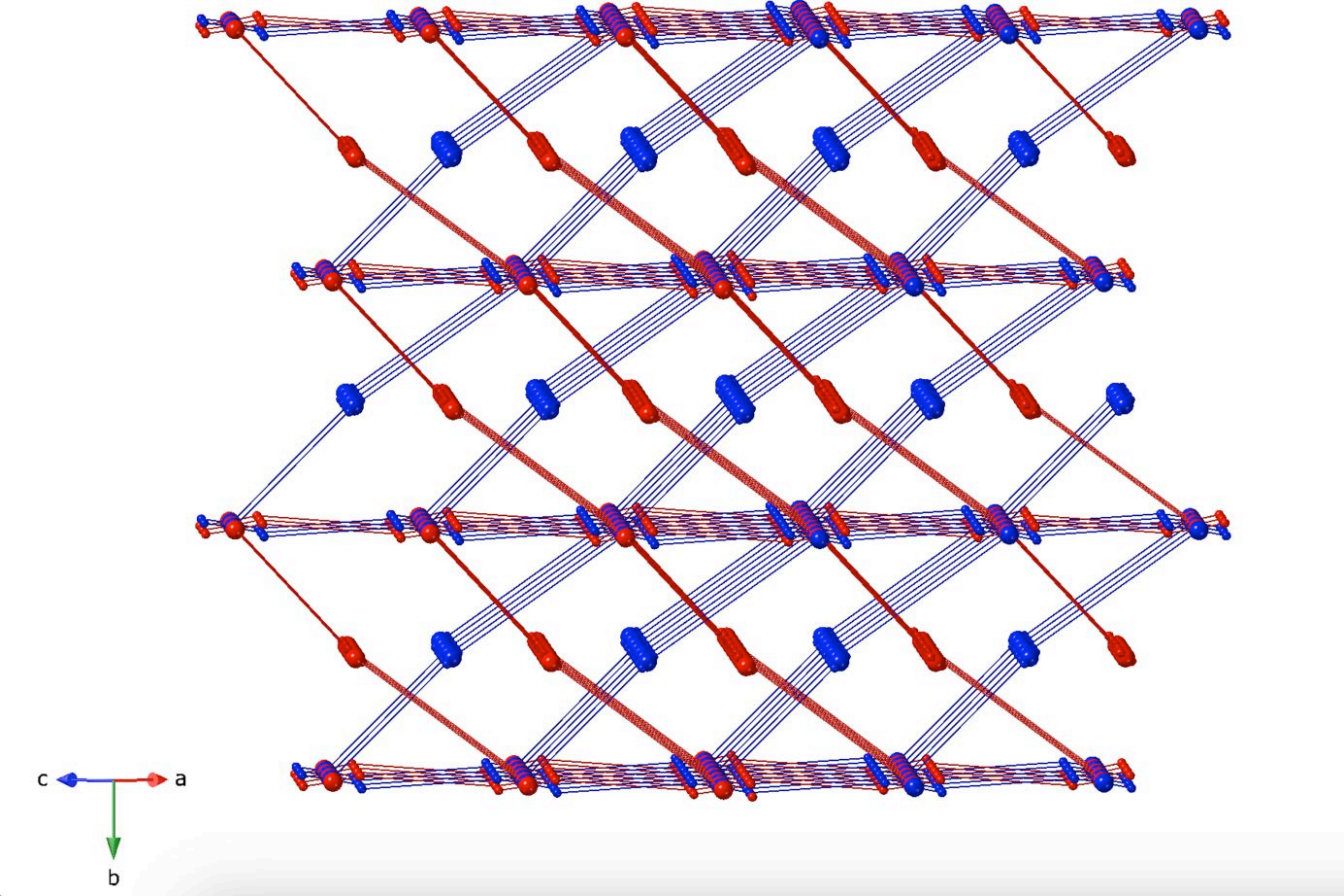
Twofold inter­penetration of **cds** topology networks in the title compound. The Cu atoms are depicted as 4-connected nodes. The rods represent through-ligand contacts between Cu atom nodes.

The ceb ligands bridge adjacent copper atoms in a bis­(monodentate) fashion to construct [Cu(ceb)]_*n*_ mono-periodic chain submotifs arranged parallel to [101] and [ 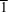 01], in which the Cu⋯Cu inter­nuclear distance measures 12.858 (2) Å (Fig. 2 ▸). These chain motifs are connected into a [Cu(ceb)(bpmp)]_*n*_ 6^5^8 topology **cds** (Blatov *et al.*, 2014 ▸) coordination polymer tri-periodic network (Fig. 3 ▸). The through-ligand Cu⋯Cu inter­nuclear distance across a bpmp ligands measures 16.406 (2) Å. Incipient void space within a single [Cu(ceb)(bpmp)]_*n*_ network allows inter­penetration of an additional network to instill a twofold system of inter­penetrated networks in the title compound (Fig. 4 ▸). A schematic perspective of the twofold inter­penetrated **cds** topology is depicted in Fig. 5 ▸. The water mol­ecules of crystallization lie in small pockets within the twofold inter­penetrated coordination polymer networks. While the H atoms of the disordered water mol­ecules of crystallization could not be found or reliably calculated, inferences can be drawn about hydrogen-bonding contacts. The major disorder component water mol­ecule O1*W* engages in hydrogen-bonding to the ceb major component O3 atom [O⋯O distance = 3.023 (1) Å]. The minor disorder component water mol­ecule O2*W* is weakly inter­acting with Cu1*A* [3.439 (1) Å], and engages in hydrogen-bonding donation to the unligated minor disorder component O4*A* atom within the ceb ligands (O⋯O distance = 2.856 Å).

## Synthesis and crystallization

Cu(NO_3_)_2_·2.5H_2_O (86 mg, 0.37 mmol), 4-(carb­oxy­eth­yl)benzoic acid (cebH_2_) (72 mg, 0.37 mmol), 1,4-bis­(pyridin-4-ylmeth­yl)piperazine (bpmp) (99 mg, 0.37 mmol), and 0.75 ml of a 1.0 *M* NaOH solution were placed into 10 ml of distilled water in a Teflon-lined acid digestion bomb. The bomb was sealed and heated in an oven at 393 K for 48 h, and then cooled slowly to 273 K. Green crystals of the title complex were obtained in 52% yield.

## Refinement

Crystal data, data collection and structure refinement details are summarized in Table 2 ▸. All H atoms attached to C were placed in calculated positions and refined with a riding model. The H atoms of the disordered water mol­ecules of crystallization could not be found from difference-Fourier maps, and attempts to use calculated positions did not give chemically reasonable inter­actions. Disorder of the Cu^II^ atoms, water mol­ecules of crystallization and ceb ligands was found and refined in a 0.655 (6):0.345 (6) ratio for all disorder components. EADP commands were used to restrain the atomic displacement parameters for the disordered components. Without these restraints, substantial numbers of non-positive definite ADPs occurred. In addition, DFIX commands were used to restrain bond lengths within the disordered parts of the ceb ligands. Otherwise, unreasonable bond lengths were occurring.

## Supplementary Material

Crystal structure: contains datablock(s) I, 1R. DOI: 10.1107/S2414314623008556/wm4198sup1.cif

Structure factors: contains datablock(s) I. DOI: 10.1107/S2414314623008556/wm4198Isup2.hkl

CCDC reference: 2298035

Additional supporting information:  crystallographic information; 3D view; checkCIF report

## Figures and Tables

**Table 1:** Selected geometric parameters (Å, °)

Cu1—O1	1.900 (9)	Cu1—N1^ii^	2.033 (11)
Cu1—O3^i^	2.069 (19)	Cu1—N4	2.021 (10)
			
O1—Cu1—O3^i^	177.2 (7)	N1^ii^—Cu1—O3^i^	82.5 (6)
O1—Cu1—N1^ii^	95.3 (4)	N4—Cu1—O3^i^	94.5 (6)
O1—Cu1—N4	87.9 (4)	N4—Cu1—N1^ii^	173.1 (6)

Symmetry codes: (i) 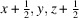 ; (ii) 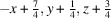 .

**Table 2:** Experimental details

Crystal data	
Chemical formula	[Cu(C_10_H_8_O_4_)(C_16_H_20_N_4_)]·H_2_O
*M*_r_	542.08
Crystal system, space group	Orthorhombic, *F* *d* *d*2
Temperature (K)	173
*a*, *b*, *c* (Å)	18.402 (2), 33.377 (4), 17.963 (2)
*V* (Å^3^)	11032 (2)
*Z*	16
Radiation type	Mo *K*α
μ (mm^−1^)	0.83
Crystal size (mm)	0.20 × 0.14 × 0.12

Data collection	
Diffractometer	Bruker APEXII CCD
Absorption correction	Multi-scan (*SADABS*; Krause *et al.*, 2015 ▸)
*T*_min_, *T*_max_	0.663, 0.745
No. of measured, independent and observed [*I* > 2σ(*I*)] reflections	21363, 5083, 3868
*R*_int_	0.060
(sin θ/λ)_max_ (Å^−1^)	0.604

Refinement	
*R*[*F* ^2^ > 2σ(*F* ^2^)], *wR*(*F* ^2^), *S*	0.103, 0.302, 1.16
No. of reflections	5083
No. of parameters	323
No. of restraints	10
H-atom treatment	H-atom parameters constrained
Δρ_max_, Δρ_min_ (e Å^−3^)	2.09, −0.67
Absolute structure	Flack *x* determined using 1453 quotients [(*I* ^+^)−(*I* ^−^)]/[(*I* ^+^)+(*I* ^−^)] (Parsons *et al.*, 2013 ▸)
Absolute structure parameter	0.043 (12)

Computer programs: *COSMO* (Bruker, 2009 ▸), *SAINT* (Bruker, 2014 ▸), *SHELXT* (Sheldrick, 2015*a* ▸), *SHELXL* (Sheldrick, 2015*b* ▸), *CrystalMaker X* (Palmer, 2020 ▸), and *OLEX2* (Dolomanov *et al.*, 2009 ▸).

## full crystallographic data

### Crystal data

**Table:**

[Cu(C_10_H_8_O_4_)(C_16_H_20_N_4_)]·H_2_O	*D*_x_ = 1.305 Mg m^−^^3^
*M**_r_* = 542.08	Mo *K*α radiation, λ = 0.71073 Å
Orthorhombic, *F**d**d*2	Cell parameters from 6274 reflections
*a* = 18.402 (2) Å	θ = 2.4–23.7°
*b* = 33.377 (4) Å	µ = 0.83 mm^−^^1^
*c* = 17.963 (2) Å	*T* = 173 K
*V* = 11032 (2) Å^3^	Block, green
*Z* = 16	0.20 × 0.14 × 0.12 mm
*F*(000) = 4528	

### Data collection

**Table:**

Bruker APEXII CCD diffractometer	5083 independent reflections
Radiation source: sealed tube	3868 reflections with *I* > 2σ(*I*)
Graphite monochromator	*R*_int_ = 0.060
Detector resolution: 8.36 pixels mm^-1^	θ_max_ = 25.4°, θ_min_ = 1.7°
ω scans	*h* = −22→22
Absorption correction: multi-scan (*SADABS*; Krause *et al.*, 2015)	*k* = −40→40
*T*_min_ = 0.663, *T*_max_ = 0.745	*l* = −21→21
21363 measured reflections	

### Refinement

**Table:**

Refinement on *F*^2^	Hydrogen site location: inferred from neighbouring sites
Least-squares matrix: full	H-atom parameters constrained
*R*[*F*^2^ > 2σ(*F*^2^)] = 0.103	*w* = 1/[σ^2^(*F*_o_^2^) + (0.2*P*)^2^] where *P* = (*F*_o_^2^ + 2*F*_c_^2^)/3
*wR*(*F*^2^) = 0.302	(Δ/σ)_max_ < 0.001
*S* = 1.16	Δρ_max_ = 2.09 e Å^−^^3^
5083 reflections	Δρ_min_ = −0.67 e Å^−^^3^
323 parameters	Absolute structure: Flack *x* determined using 1453 quotients [(*I*^+^)-(*I*^-^)]/[(*I*^+^)+(*I*^-^)] (Parsons *et al.*, 2013)
10 restraints	Absolute structure parameter: 0.043 (12)
Primary atom site location: dual	

### Special details

**Table:**

Experimental. Data was collected using a BRUKER CCD (charge coupled device) based diffractometer equipped with an Oxford low-temperature apparatus operating at 173 K. A suitable crystal was chosen and mounted on a nylon loop using Paratone oil. Data were measured using omega scans of 0.5° per frame for 30 s. The total number of images were based on results from the program COSMO where redundancy was expected to be 4 and completeness to 0.83Å to 100%. Cell parameters were retrieved using APEX II software and refined using SAINT on all observed reflections.Data reduction was performed using the SAINT software which corrects for Lp. Scaling and absorption corrections were applied using SADABS6 multi-scan technique, supplied by George Sheldrick. The structure was solved by the direct method using the SHELXT program and refined by least squares method on F2, SHELXL, incorporated in OLEX2.
Geometry. All esds (except the esd in the dihedral angle between two l.s. planes) are estimated using the full covariance matrix. The cell esds are taken into account individually in the estimation of esds in distances, angles and torsion angles; correlations between esds in cell parameters are only used when they are defined by crystal symmetry. An approximate (isotropic) treatment of cell esds is used for estimating esds involving l.s. planes.
Refinement. The structure was refined by Least Squares using version 2018/3 of XL (Sheldrick, 2015) incorporated in Olex2 (Dolomanov *et al.*, 2009). All non-hydrogen atoms were refined anisotropically. Hydrogen atom positions were calculated geometrically and refined using the riding model, except for the Hydrogen atom on the nitrogen atom which was found by difference Fourier methods and refined isotropically.

### Fractional atomic coordinates and isotropic or equivalent isotropic displacement parameters (Å^2^)

**Table:**

	*x*	*y*	*z*	*U*_iso_*/*U*_eq_	Occ. (<1)
Cu1	0.806479	0.374469	0.839709	0.0378 (7)	0.655 (6)
Cu1A	0.782809	0.355850	0.867081	0.071 (2)	0.345 (6)
O1	0.7211 (5)	0.3854 (3)	0.7838 (5)	0.053 (2)	
O2	0.6528 (6)	0.3441 (3)	0.8513 (6)	0.079 (3)	
O3	0.4000 (12)	0.3655 (6)	0.4014 (10)	0.088 (4)	0.655 (6)
O3A	0.370 (2)	0.3444 (12)	0.419 (2)	0.088 (4)	0.345 (6)
O4	0.3248 (11)	0.3281 (5)	0.4602 (10)	0.088 (4)	0.655 (6)
O4A	0.451 (2)	0.3931 (10)	0.3855 (18)	0.088 (4)	0.345 (6)
N1	0.9577 (7)	0.1703 (5)	0.1629 (7)	0.078 (4)	
N2	0.9419 (5)	0.2410 (3)	0.4075 (6)	0.051 (2)	
N3	0.8684 (5)	0.2411 (3)	0.5484 (5)	0.046 (2)	
N4	0.8194 (6)	0.3245 (4)	0.7776 (6)	0.058 (3)	
C1	0.9248 (10)	0.2050 (5)	0.1521 (9)	0.074 (4)	
H1	0.900203	0.210212	0.106574	0.088*	
C2	0.9265 (9)	0.2326 (5)	0.2055 (9)	0.070 (4)	
H2	0.906546	0.258284	0.195517	0.084*	
C3	0.9915 (7)	0.1599 (5)	0.2295 (11)	0.079 (5)	
H3	1.013403	0.134424	0.236456	0.094*	
C4	0.9914 (7)	0.1895 (5)	0.2867 (9)	0.069 (4)	
H4	1.015475	0.184756	0.332659	0.083*	
C5	0.9556 (7)	0.2256 (4)	0.2739 (7)	0.053 (3)	
C6	0.9558 (9)	0.2571 (4)	0.3357 (8)	0.067 (4)	
H6A	1.003685	0.270645	0.336310	0.081*	
H6B	0.918493	0.277600	0.324424	0.081*	
C7	0.9589 (7)	0.2698 (4)	0.4645 (8)	0.061 (3)	
H7A	0.929828	0.294407	0.456150	0.074*	
H7B	1.010880	0.277178	0.461018	0.074*	
C8	0.8663 (6)	0.2286 (4)	0.4167 (7)	0.049 (3)	
H8A	0.854272	0.208393	0.378374	0.059*	
H8B	0.834349	0.252099	0.408708	0.059*	
C9	0.9438 (7)	0.2544 (4)	0.5390 (8)	0.059 (3)	
H9A	0.976725	0.231599	0.549256	0.071*	
H9B	0.954363	0.275613	0.576011	0.071*	
C10	0.8514 (7)	0.2116 (4)	0.4913 (7)	0.056 (3)	
H10A	0.799549	0.203841	0.494876	0.067*	
H10B	0.881259	0.187256	0.498941	0.067*	
C11	0.8537 (8)	0.2266 (4)	0.6223 (7)	0.060 (3)	
H11A	0.893912	0.208966	0.638657	0.072*	
H11B	0.808423	0.210611	0.621971	0.072*	
C12	0.8457 (7)	0.2614 (4)	0.6771 (7)	0.049 (3)	
C13	0.8341 (8)	0.2998 (4)	0.6543 (8)	0.062 (3)	
H13	0.834361	0.305650	0.602549	0.074*	
C14	0.8217 (7)	0.3309 (4)	0.7051 (8)	0.059 (3)	
H14	0.814630	0.357323	0.686816	0.071*	
C15	0.8435 (7)	0.2544 (4)	0.7534 (8)	0.059 (3)	
H15	0.850119	0.228228	0.772971	0.070*	
C16	0.8312 (7)	0.2870 (5)	0.8004 (8)	0.063 (4)	
H16	0.831158	0.282148	0.852539	0.076*	
C17	0.6648 (9)	0.3661 (4)	0.7970 (7)	0.055 (3)	
C18	0.6035 (6)	0.3684 (3)	0.7386 (6)	0.043 (2)	
C19	0.6201 (8)	0.3838 (4)	0.6685 (7)	0.055 (3)	
H19	0.668076	0.392514	0.657570	0.066*	
C20	0.5663 (8)	0.3861 (4)	0.6146 (7)	0.059 (3)	
H20	0.578916	0.396054	0.566785	0.071*	
C21	0.5332 (6)	0.3562 (4)	0.7537 (7)	0.051 (3)	
H21	0.520479	0.345576	0.800971	0.062*	
C22	0.4800 (7)	0.3602 (4)	0.6954 (9)	0.063 (4)	
H22	0.431441	0.352185	0.705291	0.076*	
C23	0.4960 (8)	0.3749 (4)	0.6272 (8)	0.060 (4)	
C24	0.4367 (17)	0.3761 (11)	0.5699 (16)	0.088 (4)	0.655 (6)
H24A	0.441108	0.401124	0.540629	0.105*	0.655 (6)
H24B	0.389028	0.376535	0.595436	0.105*	0.655 (6)
C24A	0.453 (3)	0.380 (2)	0.556 (2)	0.088 (4)	0.345 (6)
H24C	0.402645	0.372037	0.565892	0.105*	0.345 (6)
H24D	0.452545	0.409288	0.544320	0.105*	0.345 (6)
C25	0.4394 (16)	0.3405 (8)	0.5174 (14)	0.088 (4)	0.655 (6)
H25A	0.488569	0.338504	0.495429	0.105*	0.655 (6)
H25B	0.430005	0.315634	0.545862	0.105*	0.655 (6)
C25A	0.479 (3)	0.3584 (18)	0.488 (2)	0.088 (4)	0.345 (6)
H25C	0.528335	0.367874	0.473980	0.105*	0.345 (6)
H25D	0.482043	0.329295	0.498469	0.105*	0.345 (6)
C26	0.3852 (12)	0.3442 (7)	0.4572 (13)	0.088 (4)	0.655 (6)
C26A	0.427 (2)	0.3660 (13)	0.427 (2)	0.088 (4)	0.345 (6)
O1W	0.5305 (11)	0.4190 (7)	0.4251 (10)	0.102 (6)	0.655 (6)
O2W	0.880 (2)	0.4185 (14)	0.7522 (18)	0.102 (6)	0.345 (6)

### Atomic displacement parameters (Å^2^)

**Table:**

	*U*^11^	*U*^22^	*U*^33^	*U*^12^	*U*^13^	*U*^23^
Cu1	0.0397 (11)	0.0405 (11)	0.0334 (11)	0.0099 (8)	−0.0117 (8)	−0.0126 (8)
Cu1A	0.073 (4)	0.087 (5)	0.054 (3)	0.030 (3)	−0.025 (3)	−0.030 (3)
O1	0.052 (5)	0.058 (5)	0.050 (5)	0.000 (4)	−0.012 (4)	−0.008 (4)
O2	0.092 (7)	0.095 (7)	0.048 (6)	0.024 (6)	−0.013 (5)	0.015 (5)
O3	0.101 (8)	0.092 (7)	0.071 (6)	0.052 (6)	−0.046 (6)	−0.035 (5)
O3A	0.101 (8)	0.092 (7)	0.071 (6)	0.052 (6)	−0.046 (6)	−0.035 (5)
O4	0.101 (8)	0.092 (7)	0.071 (6)	0.052 (6)	−0.046 (6)	−0.035 (5)
O4A	0.101 (8)	0.092 (7)	0.071 (6)	0.052 (6)	−0.046 (6)	−0.035 (5)
N1	0.071 (8)	0.115 (11)	0.048 (7)	−0.047 (8)	0.023 (6)	−0.049 (7)
N2	0.061 (6)	0.047 (5)	0.045 (5)	−0.007 (4)	0.009 (4)	−0.017 (4)
N3	0.046 (5)	0.041 (5)	0.052 (5)	−0.002 (4)	0.005 (4)	−0.015 (4)
N4	0.053 (6)	0.075 (7)	0.046 (6)	0.023 (5)	−0.012 (5)	−0.023 (5)
C1	0.091 (11)	0.076 (10)	0.054 (8)	−0.018 (9)	0.004 (7)	−0.011 (8)
C2	0.065 (8)	0.076 (9)	0.070 (10)	−0.009 (7)	0.000 (7)	0.004 (8)
C3	0.040 (6)	0.080 (9)	0.116 (14)	−0.008 (6)	0.023 (8)	−0.048 (10)
C4	0.056 (7)	0.081 (9)	0.070 (9)	0.002 (7)	0.000 (7)	−0.045 (8)
C5	0.061 (7)	0.048 (6)	0.049 (7)	−0.001 (5)	0.014 (6)	−0.019 (5)
C6	0.088 (10)	0.051 (7)	0.063 (9)	−0.001 (6)	0.021 (7)	−0.016 (6)
C7	0.057 (7)	0.060 (8)	0.067 (9)	−0.013 (6)	0.014 (6)	−0.026 (6)
C8	0.048 (6)	0.052 (6)	0.048 (6)	−0.009 (5)	−0.007 (5)	−0.018 (5)
C9	0.059 (7)	0.057 (7)	0.062 (8)	−0.003 (6)	0.004 (6)	−0.031 (6)
C10	0.052 (7)	0.058 (7)	0.057 (7)	−0.011 (6)	0.006 (6)	−0.029 (6)
C11	0.072 (9)	0.060 (8)	0.048 (7)	−0.002 (6)	0.013 (6)	−0.020 (6)
C12	0.053 (6)	0.050 (6)	0.045 (6)	0.006 (5)	−0.001 (5)	−0.015 (5)
C13	0.074 (9)	0.060 (8)	0.051 (7)	0.018 (6)	−0.011 (6)	−0.021 (6)
C14	0.060 (7)	0.058 (7)	0.059 (8)	0.011 (6)	−0.013 (6)	−0.031 (6)
C15	0.057 (7)	0.054 (7)	0.065 (9)	0.000 (6)	0.000 (6)	−0.016 (6)
C16	0.050 (7)	0.095 (11)	0.044 (7)	0.012 (7)	−0.015 (5)	−0.024 (7)
C17	0.087 (10)	0.048 (6)	0.030 (6)	0.023 (6)	0.001 (6)	−0.010 (5)
C18	0.053 (6)	0.039 (5)	0.036 (6)	0.013 (5)	−0.011 (5)	−0.008 (4)
C19	0.063 (8)	0.064 (7)	0.039 (6)	−0.004 (6)	−0.008 (6)	−0.005 (5)
C20	0.078 (10)	0.063 (7)	0.037 (6)	0.014 (6)	−0.019 (6)	0.001 (5)
C21	0.047 (6)	0.055 (7)	0.052 (7)	−0.004 (5)	0.003 (5)	0.001 (5)
C22	0.043 (6)	0.072 (8)	0.074 (9)	−0.012 (6)	−0.006 (6)	−0.027 (7)
C23	0.062 (8)	0.061 (8)	0.056 (8)	0.023 (6)	−0.029 (6)	−0.025 (6)
C24	0.101 (8)	0.092 (7)	0.071 (6)	0.052 (6)	−0.046 (6)	−0.035 (5)
C24A	0.101 (8)	0.092 (7)	0.071 (6)	0.052 (6)	−0.046 (6)	−0.035 (5)
C25	0.101 (8)	0.092 (7)	0.071 (6)	0.052 (6)	−0.046 (6)	−0.035 (5)
C25A	0.101 (8)	0.092 (7)	0.071 (6)	0.052 (6)	−0.046 (6)	−0.035 (5)
C26	0.101 (8)	0.092 (7)	0.071 (6)	0.052 (6)	−0.046 (6)	−0.035 (5)
C26A	0.101 (8)	0.092 (7)	0.071 (6)	0.052 (6)	−0.046 (6)	−0.035 (5)
O1W	0.087 (11)	0.154 (16)	0.065 (9)	0.046 (10)	−0.019 (8)	−0.021 (10)
O2W	0.087 (11)	0.154 (16)	0.065 (9)	0.046 (10)	−0.019 (8)	−0.021 (10)

### Geometric parameters (Å, º)

**Table:**

Cu1—O1	1.900 (9)	C8—C10	1.481 (18)
Cu1—O3^i^	2.069 (19)	C9—H9A	0.9900
Cu1—N1^ii^	2.033 (11)	C9—H9B	0.9900
Cu1—N4	2.021 (10)	C10—H10A	0.9900
Cu1A—O1	2.121 (9)	C10—H10B	0.9900
Cu1A—O3A^i^	1.89 (3)	C11—H11A	0.9900
Cu1A—N1^ii^	2.309 (15)	C11—H11B	0.9900
Cu1A—N4	2.033 (10)	C11—C12	1.529 (16)
Cu1A—C17	2.534 (15)	C12—C13	1.362 (18)
O1—C17	1.241 (19)	C12—C15	1.392 (19)
O2—C17	1.243 (16)	C13—H13	0.9500
O3—C26	1.26 (2)	C13—C14	1.400 (17)
O3A—C26A	1.29 (3)	C14—H14	0.9500
O4—C26	1.24 (2)	C15—H15	0.9500
O4A—C26A	1.25 (3)	C15—C16	1.395 (18)
N1—C1	1.32 (2)	C16—H16	0.9500
N1—C3	1.39 (3)	C17—C18	1.541 (17)
N2—C6	1.420 (17)	C18—C19	1.394 (17)
N2—C7	1.441 (16)	C18—C21	1.383 (17)
N2—C8	1.461 (16)	C19—H19	0.9500
N3—C9	1.467 (16)	C19—C20	1.386 (18)
N3—C10	1.457 (13)	C20—H20	0.9500
N3—C11	1.438 (16)	C20—C23	1.37 (2)
N4—C14	1.321 (19)	C21—H21	0.9500
N4—C16	1.33 (2)	C21—C22	1.441 (19)
C1—H1	0.9500	C22—H22	0.9500
C1—C2	1.33 (2)	C22—C23	1.35 (2)
C2—H2	0.9500	C23—C24	1.50 (2)
C2—C5	1.36 (2)	C23—C24A	1.51 (3)
C3—H3	0.9500	C24—H24A	0.9900
C3—C4	1.426 (19)	C24—H24B	0.9900
C4—H4	0.9500	C24—C25	1.52 (2)
C4—C5	1.39 (2)	C24A—H24C	0.9900
C5—C6	1.531 (17)	C24A—H24D	0.9900
C6—H6A	0.9900	C24A—C25A	1.50 (3)
C6—H6B	0.9900	C25—H25A	0.9900
C7—H7A	0.9900	C25—H25B	0.9900
C7—H7B	0.9900	C25—C26	1.48 (2)
C7—C9	1.46 (2)	C25A—H25C	0.9900
C8—H8A	0.9900	C25A—H25D	0.9900
C8—H8B	0.9900	C25A—C26A	1.48 (3)
			
O1—Cu1—O3^i^	177.2 (7)	C8—C10—H10B	109.7
O1—Cu1—N1^ii^	95.3 (4)	H10A—C10—H10B	108.2
O1—Cu1—N4	87.9 (4)	N3—C11—H11A	109.5
N1^ii^—Cu1—O3^i^	82.5 (6)	N3—C11—H11B	109.5
N4—Cu1—O3^i^	94.5 (6)	N3—C11—C12	110.9 (11)
N4—Cu1—N1^ii^	173.1 (6)	H11A—C11—H11B	108.1
O1—Cu1A—N1^ii^	81.9 (4)	C12—C11—H11A	109.5
O1—Cu1A—C17	29.3 (4)	C12—C11—H11B	109.5
O3A^i^—Cu1A—O1	153.4 (16)	C13—C12—C11	122.5 (12)
O3A^i^—Cu1A—N1^ii^	87.0 (11)	C13—C12—C15	116.7 (11)
O3A^i^—Cu1A—N4	90.2 (11)	C15—C12—C11	120.6 (11)
O3A^i^—Cu1A—C17	176.2 (12)	C12—C13—H13	119.1
N1^ii^—Cu1A—C17	96.6 (4)	C12—C13—C14	121.8 (13)
N4—Cu1A—O1	81.9 (4)	C14—C13—H13	119.1
N4—Cu1A—N1^ii^	137.4 (6)	N4—C14—C13	121.8 (14)
N4—Cu1A—C17	87.8 (4)	N4—C14—H14	119.1
C17—O1—Cu1	119.3 (9)	C13—C14—H14	119.1
C17—O1—Cu1A	94.1 (8)	C12—C15—H15	121.0
C26—O3—Cu1^iii^	109.1 (18)	C16—C15—C12	118.0 (13)
Cu1^iv^—N1—Cu1A^iv^	22.93 (17)	C16—C15—H15	121.0
C1—N1—Cu1^iv^	120.4 (12)	N4—C16—C15	124.8 (13)
C1—N1—Cu1A^iv^	143.0 (11)	N4—C16—H16	117.6
C1—N1—C3	123.3 (12)	C15—C16—H16	117.6
C3—N1—Cu1^iv^	115.2 (12)	O1—C17—Cu1A	56.6 (7)
C3—N1—Cu1A^iv^	92.4 (11)	O1—C17—C18	117.0 (11)
C6—N2—C7	110.6 (10)	O2—C17—Cu1A	71.4 (8)
C6—N2—C8	112.4 (11)	O2—C17—O1	127.2 (13)
C7—N2—C8	108.3 (9)	O2—C17—C18	115.7 (13)
C9—N3—C10	109.1 (9)	C18—C17—Cu1A	166.3 (8)
C11—N3—C9	112.7 (10)	C19—C18—C17	118.2 (11)
C11—N3—C10	112.5 (10)	C21—C18—C17	122.4 (11)
C14—N4—Cu1	114.5 (10)	C21—C18—C19	119.3 (11)
C14—N4—Cu1A	135.0 (10)	C18—C19—H19	120.2
C14—N4—C16	116.7 (11)	C20—C19—C18	119.7 (13)
C16—N4—Cu1	128.5 (9)	C20—C19—H19	120.2
C16—N4—Cu1A	107.0 (9)	C19—C20—H20	118.5
N1—C1—H1	120.3	C23—C20—C19	123.1 (13)
N1—C1—C2	119.3 (16)	C23—C20—H20	118.5
C2—C1—H1	120.3	C18—C21—H21	121.1
C1—C2—H2	118.6	C18—C21—C22	117.8 (12)
C1—C2—C5	122.8 (15)	C22—C21—H21	121.1
C5—C2—H2	118.6	C21—C22—H22	118.5
N1—C3—H3	121.7	C23—C22—C21	123.0 (12)
N1—C3—C4	116.6 (15)	C23—C22—H22	118.5
C4—C3—H3	121.7	C20—C23—C24	125 (2)
C3—C4—H4	120.7	C20—C23—C24A	109 (3)
C5—C4—C3	118.7 (15)	C22—C23—C20	117.1 (11)
C5—C4—H4	120.7	C22—C23—C24	118 (2)
C2—C5—C4	119.0 (12)	C22—C23—C24A	134 (3)
C2—C5—C6	122.5 (13)	C23—C24—H24A	109.1
C4—C5—C6	118.2 (12)	C23—C24—H24B	109.1
N2—C6—C5	113.4 (11)	C23—C24—C25	112.4 (18)
N2—C6—H6A	108.9	H24A—C24—H24B	107.9
N2—C6—H6B	108.9	C25—C24—H24A	109.1
C5—C6—H6A	108.9	C25—C24—H24B	109.1
C5—C6—H6B	108.9	C23—C24A—H24C	107.9
H6A—C6—H6B	107.7	C23—C24A—H24D	107.9
N2—C7—H7A	109.2	H24C—C24A—H24D	107.2
N2—C7—H7B	109.2	C25A—C24A—C23	118 (3)
N2—C7—C9	112.0 (11)	C25A—C24A—H24C	107.9
H7A—C7—H7B	107.9	C25A—C24A—H24D	107.9
C9—C7—H7A	109.2	C24—C25—H25A	109.3
C9—C7—H7B	109.2	C24—C25—H25B	109.3
N2—C8—H8A	109.0	H25A—C25—H25B	108.0
N2—C8—H8B	109.0	C26—C25—C24	111.6 (17)
N2—C8—C10	112.8 (10)	C26—C25—H25A	109.3
H8A—C8—H8B	107.8	C26—C25—H25B	109.3
C10—C8—H8A	109.0	C24A—C25A—H25C	110.0
C10—C8—H8B	109.0	C24A—C25A—H25D	110.0
N3—C9—C7	113.1 (11)	H25C—C25A—H25D	108.3
N3—C9—H9A	109.0	C26A—C25A—C24A	109 (3)
N3—C9—H9B	109.0	C26A—C25A—H25C	110.0
C7—C9—H9A	109.0	C26A—C25A—H25D	110.0
C7—C9—H9B	109.0	O3—C26—C25	119 (2)
H9A—C9—H9B	107.8	O4—C26—O3	118 (2)
N3—C10—C8	109.7 (10)	O4—C26—C25	123 (2)
N3—C10—H10A	109.7	O3A—C26A—C25A	121 (4)
N3—C10—H10B	109.7	O4A—C26A—O3A	129 (4)
C8—C10—H10A	109.7	O4A—C26A—C25A	110 (4)
			
Cu1—O1—C17—O2	12.4 (17)	C7—N2—C8—C10	−57.7 (13)
Cu1—O1—C17—C18	−165.4 (7)	C8—N2—C6—C5	−71.7 (14)
Cu1^iii^—O3—C26—O4	10 (3)	C8—N2—C7—C9	55.2 (15)
Cu1^iii^—O3—C26—C25	−167.8 (16)	C9—N3—C10—C8	−55.3 (13)
Cu1^iv^—N1—C1—C2	170.7 (11)	C9—N3—C11—C12	76.4 (13)
Cu1^iv^—N1—C3—C4	−169.9 (9)	C10—N3—C9—C7	55.4 (14)
Cu1—N4—C14—C13	176.7 (10)	C10—N3—C11—C12	−159.8 (10)
Cu1—N4—C16—C15	−176.3 (10)	C11—N3—C9—C7	−179.0 (10)
Cu1A—O1—C17—O2	11.7 (14)	C11—N3—C10—C8	178.9 (11)
Cu1A—O1—C17—C18	−166.0 (8)	C11—C12—C13—C14	175.3 (12)
Cu1A^iii^—O3A—C26A—O4A	−25 (8)	C11—C12—C15—C16	−175.7 (12)
Cu1A^iii^—O3A—C26A—C25A	159 (3)	C12—C13—C14—N4	−1 (2)
Cu1A^iv^—N1—C1—C2	165.8 (12)	C12—C15—C16—N4	2 (2)
Cu1A^iv^—N1—C3—C4	−171.6 (11)	C13—C12—C15—C16	−1.4 (19)
Cu1A—N4—C14—C13	−164.0 (11)	C14—N4—C16—C15	−2 (2)
Cu1A—N4—C16—C15	167.4 (11)	C15—C12—C13—C14	1 (2)
Cu1A—C17—C18—C19	−45 (4)	C16—N4—C14—C13	1 (2)
Cu1A—C17—C18—C21	136 (4)	C17—C18—C19—C20	179.8 (11)
O1—C17—C18—C19	14.4 (15)	C17—C18—C21—C22	179.2 (11)
O1—C17—C18—C21	−165.1 (10)	C18—C19—C20—C23	1 (2)
O2—C17—C18—C19	−163.6 (11)	C18—C21—C22—C23	0.6 (19)
O2—C17—C18—C21	16.9 (16)	C19—C18—C21—C22	−0.3 (17)
N1—C1—C2—C5	−6 (2)	C19—C20—C23—C22	−1 (2)
N1—C3—C4—C5	2.8 (19)	C19—C20—C23—C24	−178.5 (18)
N2—C7—C9—N3	−56.4 (14)	C19—C20—C23—C24A	−178 (3)
N2—C8—C10—N3	59.1 (13)	C20—C23—C24—C25	80 (4)
N3—C11—C12—C13	16.8 (18)	C20—C23—C24A—C25A	58 (7)
N3—C11—C12—C15	−169.2 (11)	C21—C18—C19—C20	−0.7 (18)
C1—N1—C3—C4	−2 (2)	C21—C22—C23—C20	0 (2)
C1—C2—C5—C4	6 (2)	C21—C22—C23—C24	177.6 (16)
C1—C2—C5—C6	−178.6 (14)	C21—C22—C23—C24A	176 (3)
C2—C5—C6—N2	140.9 (14)	C22—C23—C24—C25	−98 (3)
C3—N1—C1—C2	3 (2)	C22—C23—C24A—C25A	−119 (5)
C3—C4—C5—C2	−5 (2)	C23—C24—C25—C26	−174 (3)
C3—C4—C5—C6	179.9 (12)	C23—C24A—C25A—C26A	177 (6)
C4—C5—C6—N2	−44.1 (17)	C24—C25—C26—O3	82 (3)
C6—N2—C7—C9	178.9 (12)	C24—C25—C26—O4	−96 (3)
C6—N2—C8—C10	179.7 (10)	C24A—C25A—C26A—O3A	−85 (7)
C7—N2—C6—C5	167.0 (11)	C24A—C25A—C26A—O4A	98 (6)

Symmetry codes: (i) *x*+1/2, *y*, *z*+1/2; (ii) −*x*+7/4, *y*+1/4, *z*+3/4; (iii) *x*−1/2, *y*, *z*−1/2; (iv) −*x*+7/4, *y*−1/4, *z*−3/4.
